# Compact Multifunctional Wireless Capacitive Sensor System and Its Application in Icing Detection

**DOI:** 10.3390/s19245562

**Published:** 2019-12-16

**Authors:** Wolfgang Stocksreiter, Hubert Zangl

**Affiliations:** 1 Institute Electronic Engineering, University of Applied Sciences FH JOANNEUM GmbH, Graz 8020, Austria; 2 Institute for Smart Systems Technologies, Sensors and Actuators, Alpen-Adria-University, Klagenfurt 9020, Austria

**Keywords:** ice detection, capacitive sensor, wireless data transmission, planar, patch antenna, energy harvesting

## Abstract

When sensors are used for the monitoring of surfaces, for example, with respect to ice aggregation, it is of interest to have a full coverage of the surface without sensor-inherent detection gaps, so-called “blind spots”. Since the components of such a sensor, like antennas and energy harvesting, also require space on the surface, the actual, effective sensing area is usually much smaller than the total surface of the device. Consequently, with an array of such sensors, it is not possible to monitor the entire surface of an object without gaps, even if the sensors are mounted directly adjacent to each other. Furthermore, the excessive size may also prevent the application of a single sensor in space constraint situations as they occur, e.g., on aircrafts. This article investigates a sensor concept in which the electrodes of the sensors are used for both radio data transmission and energy harvesting at the same time. Thus, the wireless data transmission in the 2.45 GHz Industrial, Scientific and Medical (ISM) band is combined with the sensor electrodes and also with a photovoltaic cell for energy harvesting. The combination of sensor technology, communication, and harvesting enables a compact system and thus reduces blind spots to a minimum. In the following article, the structure and functionality of a system is described and verified by laboratory experiments.

## 1. Introduction

The task of a sensor in condition monitoring is to provide a medium from which electrical information can be measured as reliably and accurately as possible, in order to reconstruct the condition from the measurement results. Sensors can consist of only one component, e.g., a temperature measuring resistor, which transmits an analog value by wire. If the data conditioning is shifted to the sensor, more interference-resistant digital values can be transmitted instead of the more sensitive analog values. This results in an increased functionality of the sensor and requires additional energy. Power can be supplied via an internal source (battery cell), an external energy source conducted or near-field-coupled, or via a rechargeable battery, possibly in conjunction with an energy harvesting system. For the communication of the sensor with the base unit there are basically two possibilities available: wired and wireless data transmission. Depending on which application is pursued, both types have their entitlement. The wireless in connection with an energy harvesting system has the appeal that the sensor can be operated completely without cable connections. The disadvantages are the environmental influences on the radio communication and the range of data transmission, especially in outdoor applications, where there are weather-related influences on the antenna and thus on the radio range. The sensor on which this investigation is based is an energy self-sufficient system that uses capacitive sensors to detect ice formation on surfaces and transmits it wirelessly to a central unit [1,2,3]. This type of sensor requires electrodes with electronics to measure displacement currents, an analog to digital (AD) conversion including data processing, a radio chip with an antenna, a power supply, and energy harvesting. A wealth of functions that are shifted into the sensor also require corresponding physical space. Usually only the electrodes of the capacitance measurement are used for the detection of the environmental conditions. All other components required for the sensor are inactive with regard to the actual detection. The inactive sensor surface causes system-related gaps in the detection of so-called blind spots. Due to these gaps in detection, complete skin-like monitoring is not adequately provided. The avoidance of blind spots and the achievable performance is the subject of this investigation.

**Figure 1:**
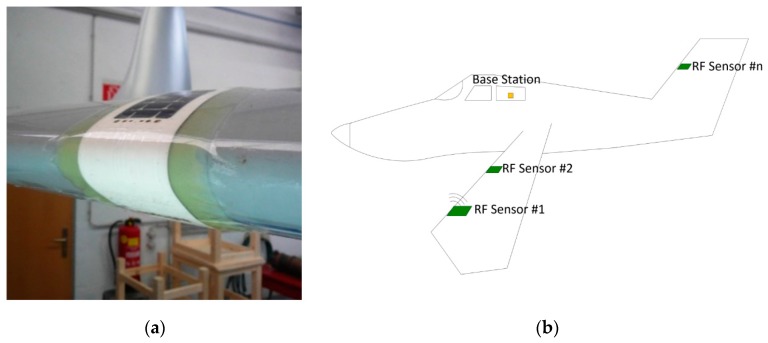
(**a**) Wireless ice sensor mounted on the outer skin of an aircraft. Photo “Wireless and Flexible Ice Detection on Aircraft” [8]. (**b**) Different exposed positions of an airplane. If ice occurs, these sensors wirelessly transmit data to the base station inside the cockpit.

Figure 1 shows a possible application for the detection of icing on an aircraft. Several sensors monitor a possible icing at different neuralgic points. The sensors can be attached in almost any density to the fuselage of the aircraft and wirelessly transmit the measured values into the cockpit of the aircraft [4,5,6,7]. The disadvantage of these sensors is that the area for energy harvesting and antenna is larger than the measuring electrodes for ice detection by a factor of 6 [8,9,10].

In addition to the detection of icing, other possible areas of application are, for example, the monitoring of structural elements on buildings with flexible thin skin-like sensors for the detection of damage such as fatigue cracks, corrosion, fractures, material fatigue, or plastic deformations [11,12,13].

The question dealt with in this context is whether it is possible to combine sensor technology, data transmission, and power generation in the concrete application in such a way that no detection gaps arise in the application. What influence do sensors and harvesting have on radio transmission and what ranges can be achieved? What is the potential to increase performance [14,15,16]? In the following, a possible hardware concept is described and the structure of the system is presented. The function of the sensor is demonstrated on a prototype. The mutual influence of radio energy generation and capacity measurement is investigated and evaluated.

## 2. Methodology

### 2.1. General Description of the Prototype

The physical principle of capacitance measurement is based on changing the capacitive coupling between the receive (RX) electrodes and the central transmit (TX) electrode by changing the effective dielectric constant due to the environmental conditions between the electrodes [17]. In the capacitive–digital converter (CDC), which is based on a sigma–delta conversion principle, the capacitance between the TX electrode and one of the four RX electrodes is determined sequentially by differential measurement [18,19,20]. The TX electrode E5 is excited via a 250 kHz signal and the resulting displacement current is measured via the RX electrode. This results in four capacitance values, which differ in their response behavior and size by the geometry of the respective RX electrodes and the distance to the TX electrode.

**Figure 2:**
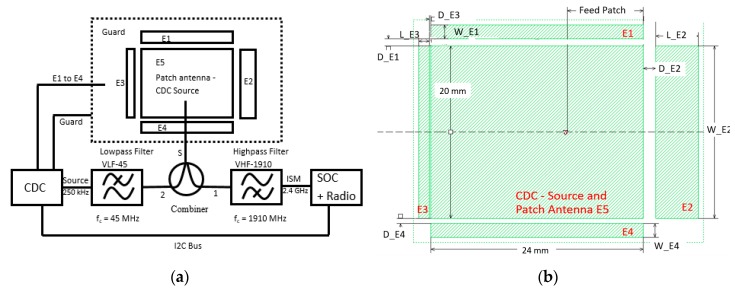
(**a**) Prototype setup with electrodes (E1 to E4), source electrode, and combined patch antenna (E5). The capacitance measurement chip (capacitive–digital-converter CDC) and processor with integrated Bluetooth radio (system-on-chip SOC). A 250 kHz source signal and 2.45 GHz radio signal combined via filter and signal combiner. (**b**) Sensor electrodes (E1 to E4) with different copper areas arranged around the source electrode and patch antenna (E5) in the middle of the configuration.

A programmable CDC AD7142 from Analog Devices with on-chip calibration was selected for the capacitance measurement [21]. The converter offered a typical capacitive input range of +/- 2 pF with 16-bit resolution and 36 ms update rate. The CDC communicated with a low-power 32-bit ARM Cortex M0 processor with an integrated 2.45 GHz radio transceiver nRF51822 from NORDIC semiconductor (Oslo, Norway) [22]. In the test setup, the common TX electrode for capacitance measurement and radio transmission was routed via a broadband 50 Ω resistive splitter and a high-pass filter to the radio section and via low-pass filters to the CDC chip (Figure 2). 

The research on the sensor system was carried out under laboratory conditions. Different capacitance to digital converters were investigated and their performance analyzed with different electrode structures [23]. 

### 2.2. Construction of the Electrode–Antenna Structure

The sensor surface was designed in such a way that it can be realized with the lowest possible installation height but still has maximum interference immunity. Three electrodes were arranged in x as well as in y alignment, with a central electrode in the middle as a capacitive transmitting electrode and simultaneously as a radio antenna (Figure 2). The other electrodes arranged around the central electrode served as receiving electrodes and were dimensioned with regard to dimensions and distances in such a way that different response behavior can be achieved with regard to the capacitance changes [24].

A patch antenna was selected as the antenna for sensors and base station. This antenna type consists of a flat ground plane on the bottom, an insulating substrate, and the conductive patch on the top. The advantage of this antenna type is that it can be built thin and the system–immanent ground plane represents a comfortable shielding and thus offers an insensitivity of the antenna to detuning due to the materials (metals, plastics) below the sensor [16,25] The thickness of the substrate, i.e., the distance between ground plane and patch, has a decisive influence on the efficiency of the radiated energy. The thickness of the substrate should not be thinner than 2% of the wavelength of the radiated frequency [26,27]. The use of materials with a higher relative epsilon reduces the propagation velocity of the radiated wave and consequently reduces the required geometric dimension for the selected resonant frequency. The disadvantage of the higher permittivity is a lower radiation efficiency due to a lower stray field at the edges of the patch. The main direction of radiation of a single patch depends on the position of the feed point with linear polarization normally standing on the patch surface. Patch antennas can be manufactured directly in the production process of a printed circuit board and therefore do not require any additional height.

The sensor antenna was built on Rogers RO3006 material with a relative epsilon of 6.5 and a favorable loss factor of 0.002, at a thickness of 640 µm and double-sided copper with 17 µm. [28] The dimensions of the patch were determined to be L = 24 mm and W = 20 mm from simulations and corresponding preliminary investigations. Due to the low substrate thickness, the bandwidth was small, but sufficient for the expected data rate. The stability of the resonant frequency as a function of temperature was significantly better with this material than with glass-reinforced epoxy laminate material (FR4), for example.

**Figure 3:**
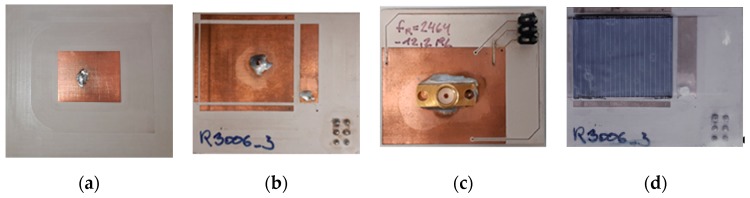
(**a**) The originally developed patch antenna (ANT1) without sensor electronics. (**b**) The patch antenna with four sensor electrodes arranged around the transmit patch. (**c**) The backside of this antenna (ANT2). All five electrodes as well as the ground electrode were guided to a pin header to either open or short the electrodes. (**d**) The antenna like ANT2, however with an insulated photovoltaic (PV) cell on the top of the central transmitting electrode named (ANT3).

Figure 3 shows the different configurations (antennas, antenna electrodes, and antenna electrode with photovoltaic cell). All three variants were based on the Rogers R3006 material, thickness 640 µm. The picture on the left shows the antenna ANT1 with full-surface ground on the back without electrodes. In the middle is the configuration ANT2 with the four measuring electrodes arranged around the antenna patch. The dimension and the feed of the antenna as well as the distance and the area of the electrodes were determined by simulation. In the middle right you can see the back of the ANT2. For shielding reasons, the ground plane extended beyond the measuring electrodes. The electrodes and the ground plane were connected to a pin header, via which the measuring electrodes could be operated either “open” (floating) or “short” (connected to ground). The antenna patch—the TX electrode for capacitance measurement—was connected via a 50 Ω coaxial Sub Miniature version A (SMA) connector.

The picture on the right shows the structure for power generation with a photovoltaic cell (PV cell) called ANT3. A monocrystalline silicon photovoltaic cell with a thickness of 250 µm and dimensions of 24 *×* 19.5 mm was used. The cell had an output of 75 mWp under standard test conditions (25 °C, AM 1.5 and 1000 W/m^2^). The electrode structure was completely insulated with 300 µm self-adhesive Polyvinylchlorid (PVC) film, so no conductive connection between antenna and photovoltaic cell was achieved. The PV cell itself was fixed with 150 µm adhesive film. Since the PV cell above the patch antenna was electrically floating and capacitively coupled with the antenna, the entire system could radiate. The PV cell caused a slight detuning of the antenna and thus mismatch losses, but no shielding.

### 2.3. Construction of the Prototype Sensor

The design of the prototype sensor consisted of a combination of two different stacked circuit board materials to improve antenna performance and temperature influences in terms of frequency stability and comparatively low dielectric losses. The upper circuit board consisted of a 640 µm thick core material of Polytetrafluorethylen (PTFE) composite materials with ceramic filling (Rogers RO3006). Only the sensor electrodes and patch antenna were located on the top layer, reducing the sensor area and minimizing blind spots. Above the TX electrode, the PV cell was positioned, insulated by plastic foil. The surface of the PV cell was exactly the same size as the patch antenna underneath. For mechanical protection and fixation, a plastic foil was attached above the PV cell. 

**Figure 4:**
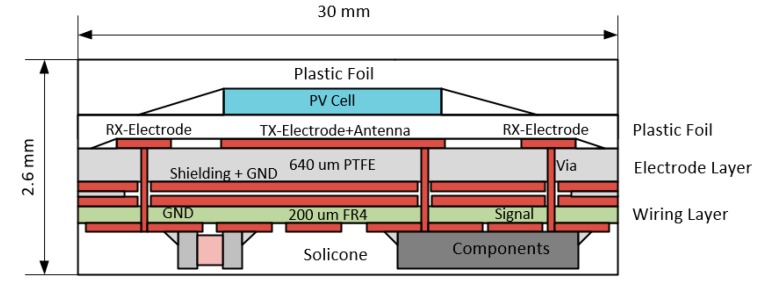
Design of the prototype sensor. The antenna and the electrode structure were on the top of the 640 µm Rogers RO3006 substrate. Electronic components and wiring were on the 200 µm FR4 substrate. The PV cell was located directly above the patch antenna. The PV cell and the electrode structure were insulated by a PVC foil.

The bottom layer of the PTFE substrate was made entirely of copper, which was used for shielding and concurrently as ground plane for the patch antenna. The electrode printed circuit board (PCB) was connected to a 200 µm thick FR-4 laminate below the Rogers material. The FR-4 PCB served as the wiring plane and as a carrier for the electronic components. This construction ensured that the ground plane, which was essential for the function of the patch antenna, was not cutted by the traces of the electrodes. In addition, the traces on the wiring level were very well shielded from above by the ground plane. The stack, including the electronic components, had a thickness of approximately 2.6 mm and an area of 30 × 30 mm (Figure 4).

The functionalities of the prototype were experimentally investigated with a near-field scanner and an anechoic chamber to determine the influence of the electrodes and the PV cell on the antenna performance. The starting point of the investigations was a patch antenna without electrodes arranged all around and without a PV cell. The changes in antenna gain, antenna matching, and transmission range were investigated.

**Figure 5:**
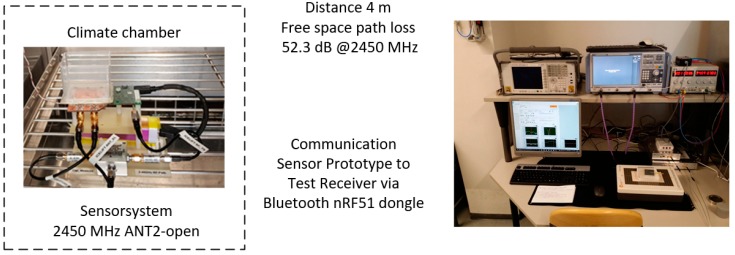
Sensor prototype inside the climate chamber, with mounted plastic foil at the sensor electrodes and a plastic water tank above. Wireless data transmission, acquisition via Bluetooth dongle nRF51 outside the chamber at a distance of 4 m.

The capacitance measurement and the influences of water and ice were tested in a climatic chamber (Figure 5) at different temperatures. Different icing scenarios were simulated. The behavior of the capacitance measurement and the radio link as a function of the temperature in the range of +25 to −40 °C was investigated. In numerous test series, water or ice of different thicknesses was applied to the sensor system and the behavior of the capacity change and the influence on the data transmission were investigated. To perform these measurements, a water tank was installed above the electrodes to precisely adjust the water level. The experiments were carried out with ice thicknesses between 1 and 10 mm. The measured capacitance values were wirelessly transmitted from the prototype sensor inside the climate chamber via Bluetooth to a computer 4 m away [29].

## 3. Results and Discussion

### 3.1. Influence of the Measurement Electrodes and the Pv-Cell on the Radio Transmission

**Figure 6:**
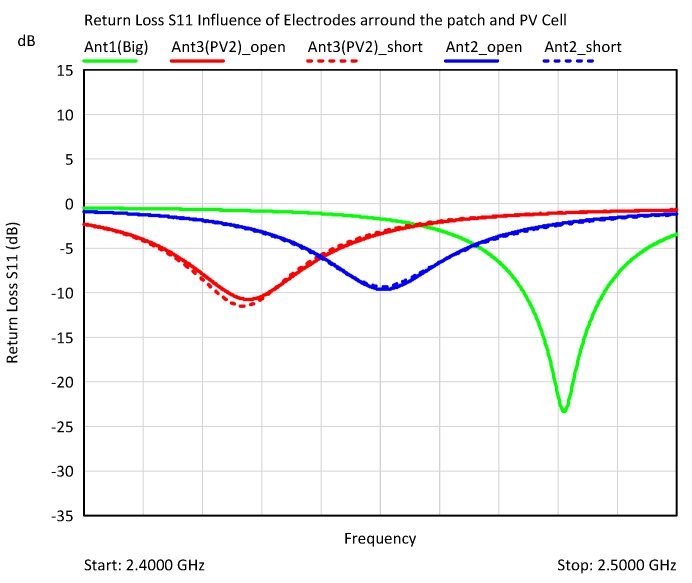
Comparison of the return losses S11 of the different antenna configurations. Green: the original antenna ANT1 with a large ground plane, without electrodes positioned around the antenna patch. Blue: the antenna with electrodes around. Red: the antenna including PV cell above the antenna patch. Solid lines: floating electrodes; dashed: shorted electrodes to ground.

The influence of the electrodes around the patch antenna and the effect of the PV cell on the return loss S11 and the resonance frequency is shown in Figure 6. The original antenna ANT1 with the antenna was compared with the electrodes arranged around the patch ANT2 and the antenna with the electrodes and the photovoltaic cell ANT3, whereby the electrodes were connected once floating and once directly to ground. 

The measurements were done with a network analyzer (N9917A Fieldfox Microwave Analyzer, Keysight Technologies, 1400 Fountaingrove Parkway Santa Rosa, California, CA, USA). The electrodes caused a shift in frequency due to the additional capacitive load of the electrodes and the cell to lower frequencies.

Remarkably, there was hardly any difference between floating (solid lines) and shorted (dashed lines) electrodes in the given configuration. The PV cell caused an additional shift of the resonant frequency. However, the frequency shifts lie within a frequency range that can be corrected by changing the antenna geometry.

The floating electrodes tended to cause a lower shift of the resonant frequency than the short-circuited ones. The change of the antenna impedance by the electrodes was between 30% and 40% related to 50 Ω.

**Figure 7:**
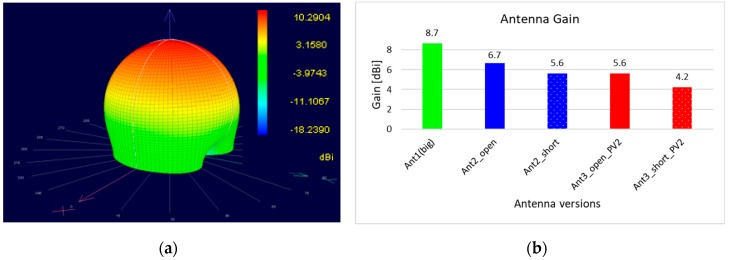
Comparison of antenna gains with and without electrodes and PV cell. (**a**) The radiation pattern of the antenna with the highest gain, without electrodes. Since the characteristic was similar for all five antennas, only the antenna with the maximum gain is displayed. The diagram (**b**) shows the antenna gain of all five antenna configurations.

A near-field scanner (RFxpert RFX, MDL Technologies, Letchworth, Hertfordshire, UK) was used to determine the differences between the antennas with respect to the resulting antenna gain. The antennas were measured at the frequency with the best matching (Figure 6). Since the patch dimensions were not changed with regard to their geometry, the resonance frequencies differed relatively clearly from each other. The antenna with electrodes was located approximately in the middle of the ISM band, the antenna without electrodes at the upper band boundary, the antenna with electrodes and PV cell far below the ISM band center. The electrodes shifted the resonant frequency of the antenna and also reduced the antenna gain; depending on whether they were floating or short-circuited, the loss of antenna gain was 2 or 3 dB. The PV cell above the antenna patch meant an additional reduction of the antenna gain by 1.3 dB. However, the influence of the electrodes as well as the one of the PV cells was relatively small. Figure 7 shows the antenna gain of the different antenna configurations.

The gain of the antenna with electrodes showed a reduction of the radiated power in the main beam direction, but a higher radiated power at the edges than with the antenna without electrodes. The electrodes caused a slight shift of the radiated power towards the edges of the antenna, therefore the maximum gain in the middle of the patch was reduced. The application of the PV cell caused an approximately uniform gain reduction over the entire antenna. The short-circuited electrodes with PV cell showed a significantly more distinctive beam in the X direction with radiation than those with floating electrodes.

**Figure 8:**
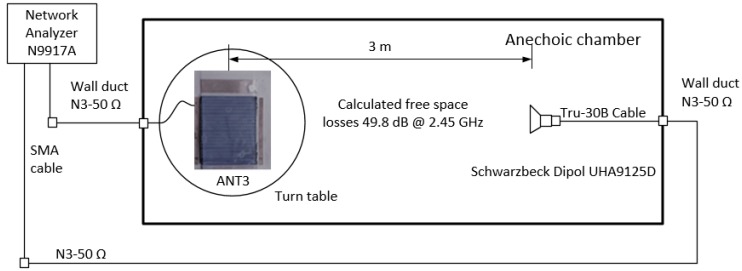
Measurement of transmission losses S21 in the anechoic chamber with a broadband dipole receive antenna (UHA9125D).

In addition to the mismatch losses due to the matching and gain reduction, the transmission losses were checked by far-field measurement in the anechoic chamber (Figure 8). A broadband dipole antenna (UHA9125D Schwarzbeck Mess-Elektronik OHG, 69250 Schönau, Germany) was used as the receiving antenna.

**Figure 9:**
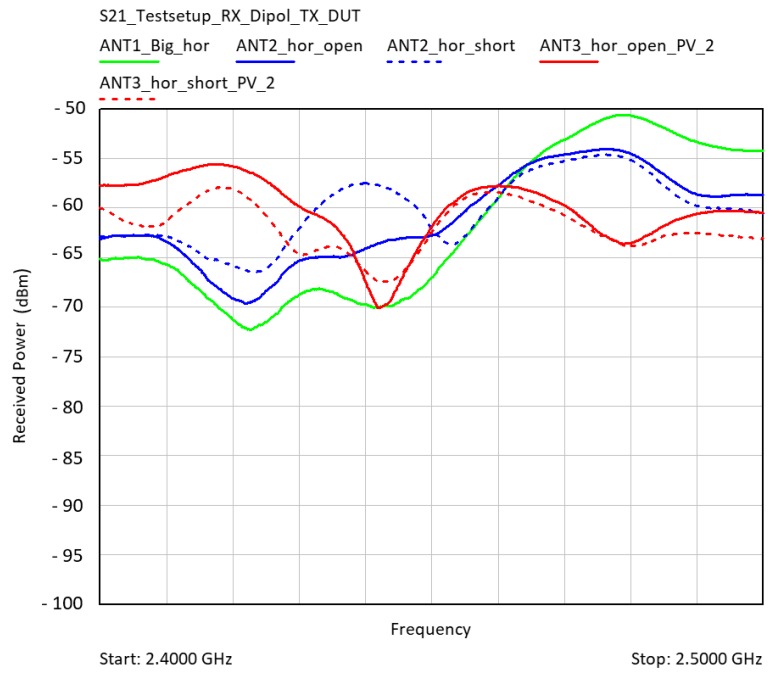
Received power at the Schwarzbeck dipole antenna in horizontal antenna orientation. Green: ANT1 with only the patch antenna; blue: ANT2 with electrodes; and red: ANT3 with PV-Cell. 0 dBm feed power at the Device Under Test DUT. The continuous line indicates the received power with floating electrodes. The dashed line indicates the power of the shorted ones.

The used dipole had a continuous −10 dB return loss over the entire ISM band from 2.40 GHz to 2.483 GHz. The transmission losses for a distance of 3 m were calculated without multipath propagation at 2450 MHz at −49.8 dB (Equation (1)). The original patch antenna without electrodes came very close to the calculated path losses (Figure 9).

(1) $$PL=20\log\left(d\right)+20\log\left(f\right)+20\log\left(\frac{4\pi }{c}\right)\;$$

where *c* = speed of light, *d* = distance between transmit and receive antenna, and *f* = frequency.

The measured transmission losses of the antenna ANT1 were approximately in the range of the calculating losses. The influences by the electrodes and the PV cell were present, but they were in the range of 5 dB. This means that gain, return loss, resonant frequency, and transmission losses were influenced, but were of a controllable size, so that they could be optimized by adjusting the patch geometry, the antenna feed, and a matching network. The next section examines whether a capacitance measurement is possible with the defined electrode structure.

### 3.2. Capacitance Measurement

**Figure 10:**
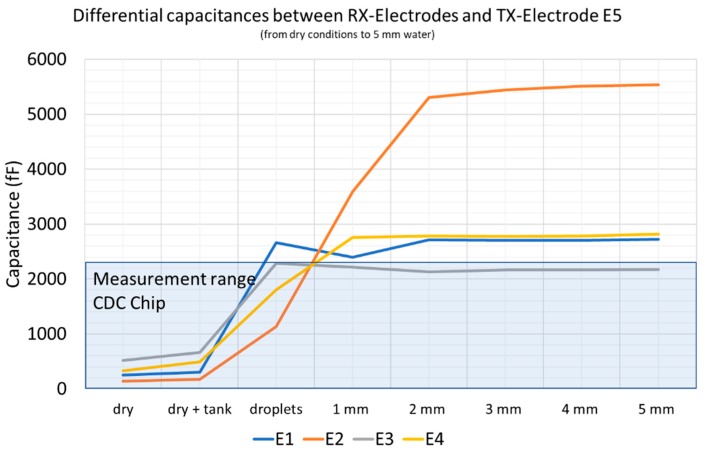
Measured capacitances between the transmitting electrode E5 and the four receiving electrodes E1 to E4. The values were measured with an LCR bridge. If there was moisture on the electrodes, the capacitance increased rapidly. The blue colored area represents the measuring range of the CDC used.

The investigation of wireless data transmission has indeed influenced the performance, but within a controllable range. In a second series of tests, the function of ice detection was investigated. Figure 10 shows the resulting capacitances from the electrode configuration. These are differential capacitances between E5 and the four receiving electrodes E1 to E4 measured by a LCR bridge 3533-01 (Hioki E.E. Corporation, 81 Koizumi Ueda, Nagano 386–1192 Japan). 

In the dry state at room temperature, capacitances between C = 150 and C = 600 fF were measured, depending on the electrode geometry and the distance to the transmit electrode E5. If there were water drops or a continuous water film over the electrodes, the capacitances increased to values between 2000 and 5000 fF. These capacitance values were already set at a water height of 2 mm and did not change noticeably at higher water levels. The area shown in blue is the measuring range of the selected CDC. In dry conditions, the measured capacitance was in the lower quarter of the measuring range. If there were drops of water or a water film on the electrodes, the measuring range was quickly exceeded and the resulting capacitance reached a geometry-related saturation capacitance.

**Figure 11:**
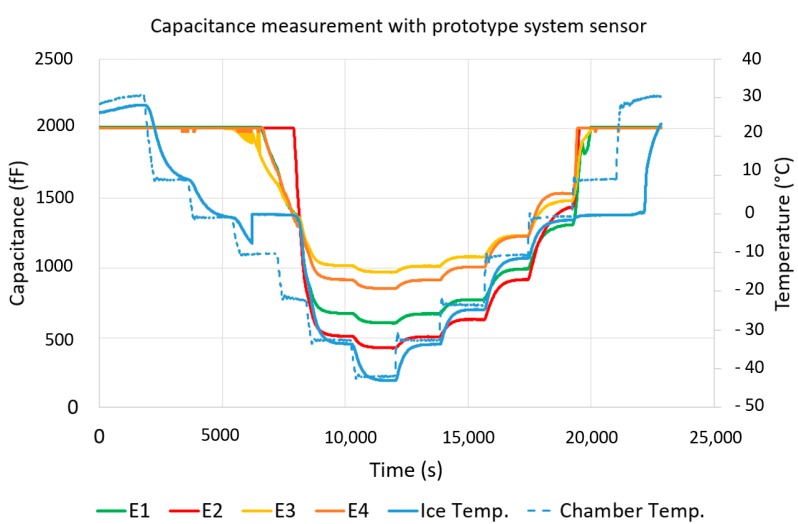
Measured differential capacitances of the prototype system between the receive electrodes E1 to E4 and the transmit electrode E5 depending on the temperature between +30 and −40 °C at a water level of 3 mm.

The capacitance values measured with the prototype system are shown in Figure 11. The starting point was covering the electrodes with 3 mm water. When reducing the temperature and freezing water, the capacitance values were set within the CDC measuring range. Beginning from the phase transition from water to ice, the capacitance values could be measured, which ranged from 1200 to 1500 fF at 0 °C. If the temperature was further reduced, the capacitance decreased to values between 400 and 900 fF at −40 °C. When the ice melted, meaning when the temperature rose, the capacitance change was significantly higher than in the opposite direction. The reason for this is the formation of a water film between the electrodes and the ice layer. During the entire test, the measurement data were transmitted wirelessly to the evaluation electronics without interruption at intervals of one second [18,19,20,30].

The selected Teflon material as a carrier for the electrodes had a thermal expansion coefficient of 17 ppm/°C in the X–Y direction. The resulting change in length was approximately 1 µm at 65 K temperature change. With a minimum electrode distance of 200 µm, the influence on the capacitance was in the range of 5‰ in the worst case. A special erosion protection film (3M W8607), also used on wind turbine rotor blades, was used to protect the prototype sensor from mechanical damage and direct contact of the electrodes with ice and water. In order to prevent condensation in the sensor, the film was glued flat over the electrodes without air inclusions. Capacitance measurements with increased air humidity caused an earlier saturation, as soon as condensation and ice formation occurred at the electrodes, the resulting capacitance was within the measuring range of the CDC (Figure 11).

The noise floor of the capacitance measurement depended on the inherent noise of the CDC and the configuration of the electrodes. At room temperature the noise floor was in the range of 130 fF. The output noise (peak to peak) of the CDC was about 20 fF at room temperature and had a decimation rate of 128 according to the data sheet. The signal to be measured was between 200 and 600 fF at room temperature, at ice and 0 °C ambient temperature it was in the range of 500 to 1200 fF.

With the prototype sensor it could be proven that it is possible to detect icing, the measured capacitances of all four electrodes were within the measuring range of the CDC. Before the formation of ice and during the defrosting phase, the capacity values increased and the measuring range was exceeded. The radio communication was given during the entire test series at a distance of 4 m. The maximum achievable range was not determined in this test setup.

### 3.3. Environmental Influences on the Radio Transmission

As shown in previous sections, the influence of the measuring electrodes and also the PV cell on the radio performance was within a controllable range, even the detection of ice was reliably feasible. In the third test series, the detuning of the antenna by environmental influences such as water and ice was investigated [16,20].

**Figure 12:**
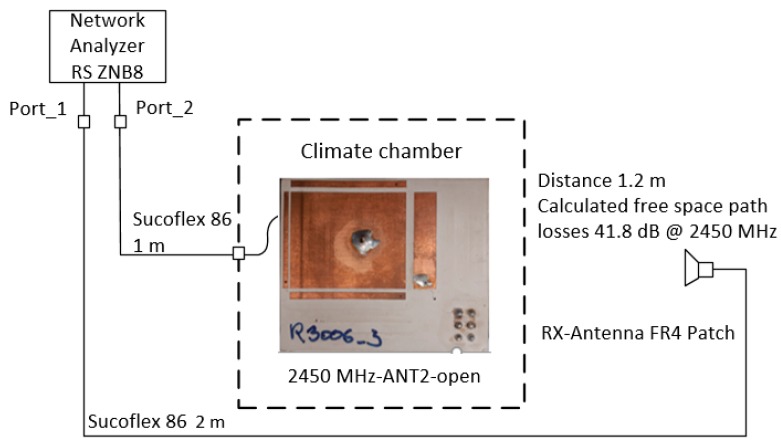
Measurement setup in the climatic chamber to investigate the environmental influences on the antenna of the sensor system. Inside the chamber there was the sensor prototype antenna, and at a distance of 1.2 m there was a FR4 patch antenna. Transmission S21 and return loss S11 were measured with a network analyzer.

The starting point of the investigations was the optimized antenna ANT2 at 2450 MHz with surrounding electrodes in the climatic chamber. Using a network analyzer, the return loss S11 and the transmission losses S21 to a receiving antenna at a distance of d = 1.2 m outside the climatic chamber were observed (Figure 12).

The temperature influence on dry electrodes showed a decrease in the resonance frequency from 2450 MHz at 25 °C to 2433 MHz at −10 °C without changing the antenna impedance. The antenna optimized for 25 °C reduced its return loss, resulting in a calculated mismatch loss.

The impedance of the antenna remained unchanged; it only experienced a frequency shift of approximately 15 MHz in the given case. This behaviour could be observed to be more pronounced in the antennas with FR4 material, whereby the frequency shift was accompanied by a change in the impedance or reflection factor.

In contrary, influences like wetness (continuous water film) and ice, as well as melting ice, at the antenna patch led to a frequency shift and to reductions of the return loss respective to an increase of the signal reflection at the antenna, i.e., the antenna impedance and the resonance frequency changed. 

**Figure 13:**
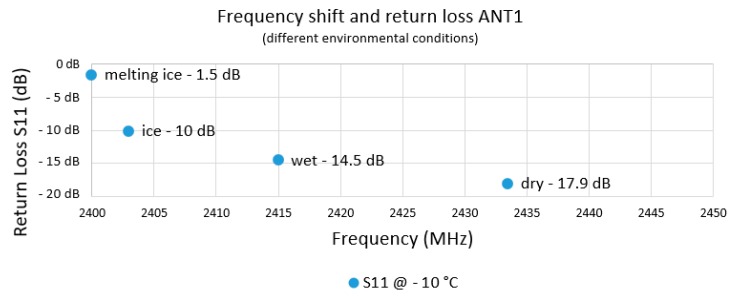
Frequency and impedance of the antenna shifted as a function of environmental influences. Melting ice had the greatest influence on the radio performance.

Figure 13 shows the shift of the resonant frequency as well as the return loss, depending on the environmental conditions. In the dry state, the return loss at −10 °C was −17.9 dB at a resonant frequency of 2435 MHz. The influences on the radio communication at melting ice were very unfavorable. The frequency shift was more than 40 MHz and was thus outside the lower ISM band limit of 2400 MHz [31], there was an almost total reflection of the radio signal at the antenna with only −1.5 dB return loss.

The temperature cycles and the associated change from frost to dew conditions had an influence on the mechanical expansion of the carrier material, copper tracks, plated-through holes, and solder connections. The different coefficients of thermal expansion were taken into account in the design of the layout. With similar structures, a durability of several years is achievable [8].

## 4. Conclusions and Outlook

The present work dealt with the investigation of a novel capacitive sensor concept in which the sensor electrodes, the radio antenna, and the energy harvesting were combined in such a way that, as far as possible, no detection gaps, so-called blind spots, arose, as they usually arise with conventional construction. A sensor prototype was built, which had only the measuring and transmitting electrodes and a PV cell on its surface and therefore no inactive sensor area. The presented prototype was investigated on the basis of an application for the detection of icing on surfaces, for example on wings of airplanes or wind turbines [32]. A planar capacitive sensor system was selected for the detection of icing, consisting of a transmitting electrode and four receiving electrodes arranged around this electrode. The capacitance built up between the electrodes was measured differentially and the capacitance changes resulting from ice formation were evaluated. It could be experimentally proven that the sensor design works in a temperature range from 25 to −40 °C and the measured capacitance values can be transmitted wirelessly via Bluetooth. The influence of the PV cell directly above the patch antenna caused a shift in the resonance frequency and a change in the antenna impedance. However, this influence is of a manageable magnitude and can be reduced by optimizing the antenna geometry and using a matching network.

The optimization potential of the prototype arises from the tuning of the receiving electrodes in order to reach the capacitive saturation range somewhat later and still remain sensitive enough. The transmitter range can be increased by optimizing the antenna path, especially the filter and splitter, and by improving the tuning of the transmitter electrode.

## 5. Patents

Patent application A 264/2018 Wolfgang Stocksreiter, Hubert Zangl “Combined sensor and communication device” for examination by the patent office Vienna.
